# Supplementary material for: Arabic Wikipedia users’ personalized behavior analysis considering gender gap
Source: PLoS One. 2024 Oct 21;19(10):e0312176. doi: 10.1371/journal.pone.0312176 (PMC11493248; doi:10.1371/journal.pone.0312176)
Supplement: Attachment — Submitted filename: responses.docx [file pone.0312176.s001.docx]

Dear Dr. Omar,

Thank you for the valuable comments that we have received to enhance the quality of our research article. Following, please find our responses to the comments we have received, in RED, and their reflections on the resubmitted paper highlighted in yellow.

Best Regards,

Bashar

**Journal requirements:**

When submitting your revision, we need you to address these additional requirements.

1. Please ensure that your manuscript meets PLOS ONE's style requirements, including those for file naming. The PLOS ONE style templates can be found at

<https://journals.plos.org/plosone/s/file?id=wjVg/PLOSOne_formatting_sample_main_body.pdf> and

<https://journals.plos.org/plosone/s/file?id=ba62/PLOSOne_formatting_sample_title_authors_affiliations.pdf>

I have tried to fulfil all the requirements as stated in the aforementioned documents and on the website. If I missed anything please let me know and I will fix it as soon as possible.

2. We suggest you thoroughly copyedit your manuscript for language usage, spelling, and grammar. If you do not know anyone who can help you do this, you may wish to consider employing a professional scientific editing service.

Whilst you may use any professional scientific editing service of your choice, PLOS has partnered with both American Journal Experts (AJE) and Editage to provide discounted services to PLOS authors. Both organizations have experience helping authors meet PLOS guidelines and can provide language editing, translation, manuscript formatting, and figure formatting to ensure your manuscript meets our submission guidelines. To take advantage of our partnership with AJE, visit the AJE website (<http://learn.aje.com/plos/>) for a 15% discount off AJE services. To take advantage of our partnership with Editage, visit the Editage website ([www.editage.com](http://www.editage.com/)) and enter referral code PLOSEDIT for a 15% discount off Editage services.  If the PLOS editorial team finds any language issues in text that either AJE or Editage has edited, the service provider will re-edit the text for free.

Upon resubmission, please provide the following:

The name of the colleague or the details of the professional service that edited your manuscript

A copy of your manuscript showing your changes by either highlighting them or using track changes (uploaded as a *supporting information* file)

A clean copy of the edited manuscript (uploaded as the new *manuscript* file).

The manuscript was edited with the help of Editage as suggested. The editing certificate is attached with the manuscript.

3. Please change "female” or "male" to "woman” or "man" as appropriate, when used as a noun (see for instance <https://apastyle.apa.org/style-grammar-guidelines/bias-free-language/gender>).

DONE

4. In your Methods section, please include additional information about your dataset and ensure that you have included a statement specifying whether the collection and analysis method complied with the terms and conditions for the source of the data.

DONE, licensing of the collected data is highlighted in the supporting manuscript.

5. Please provide a complete Data Availability Statement in the submission form, ensuring you include all necessary access information or a reason for why you are unable to make your data freely accessible. If your research concerns only data provided within your submission, please write "All data are in the manuscript and/or supporting information files" as your Data Availability Statement.

DONE, licensing of the collected data is highlighted in the supporting manuscript.

**Additional Editor Comments:**

Dear authors, the manuscript needs major revisions, please respond point by point to the reviewers.

Kind regards.


[Note: HTML markup is below. Please do not edit.]

Reviewers' comments:

Reviewer's Responses to Questions

**Comments to the Author**

1. Is the manuscript technically sound, and do the data support the conclusions?

The manuscript must describe a technically sound piece of scientific research with data that supports the conclusions. Experiments must have been conducted rigorously, with appropriate controls, replication, and sample sizes. The conclusions must be drawn appropriately based on the data presented.

Reviewer #1: Partly

Reviewer #2: Partly

Reviewer #3: Partly

2. Has the statistical analysis been performed appropriately and rigorously?

Reviewer #1: Yes

Reviewer #2: No

Reviewer #3: No

3. Have the authors made all data underlying the findings in their manuscript fully available?

The [PLOS Data policy](http://www.plosone.org/static/policies.action#sharing) requires authors to make all data underlying the findings described in their manuscript fully available without restriction, with rare exception (please refer to the Data Availability Statement in the manuscript PDF file). The data should be provided as part of the manuscript or its supporting information, or deposited to a public repository. For example, in addition to summary statistics, the data points behind means, medians and variance measures should be available. If there are restrictions on publicly sharing data—e.g. participant privacy or use of data from a third party—those must be specified.

Reviewer #1: Yes

Reviewer #2: Yes

Reviewer #3: Yes

4. Is the manuscript presented in an intelligible fashion and written in standard English?

PLOS ONE does not copyedit accepted manuscripts, so the language in submitted articles must be clear, correct, and unambiguous. Any typographical or grammatical errors should be corrected at revision, so please note any specific errors here.

Reviewer #1: No

Reviewer #2: No

Reviewer #3: Yes

5. Review Comments to the Author

Please use the space provided to explain your answers to the questions above. You may also include additional comments for the author, including concerns about dual publication, research ethics, or publication ethics. (Please upload your review as an attachment if it exceeds 20,000 characters)

Reviewer #1: 1. The data used was downloaded online from the Wikipedia history page that summarizes actions. This however does not give information on the content to highlight topics about women.

Content of pages were not analysed. The focus was on editor profiles, whether male or female, and how they interact with Wikipedia characterized by Wikipedia predefined actions

You mentioned that the profiling topics will be discussed in terms of gender theory with a specialized input from a gender specialist to give a thorough analysis of the contributor's behavior. The results for this are missing.

True, we added a paragraph starting by line 167 discussing that. Thank you for pointing that out.

2. The introduction section mixed up many issues that include Contributors, collaborators, Editors, content & actions. The Authors should be specific on what was studied. The results are about the editing behavior in light to being male or female is this enough to give a thorough gender perspective?

Yes, we believe that paragraph starting on line 167 will clarify on this point.

3. The Abstract should be written in the (Introduction, Methods, Results and Conclusion) format.

Done

4. The study was not an experiment and therefore the subheading on Experimental Results should be revised.

Fixed to Results and discussions. It looks more appropriate. Thank you.

5. The results speaking to the average actions per year between the two genders should be presented better. Using this figure is very confusing to the person interpreting the results.

An alternative table is provided in the supporting information section (Table 3)

6. The main text of the manuscript should be written in the (Introduction/ Background, Methods, Results, Discussion and conclusion) format.

Done

7. The conclusion brings in a new term "assigned tasks" this has not been mentioned or discussed anywhere in the text.

Fixed to “tasks” only

8. In the abstract you indicated that "some administration activities are disclosed for males compared to women" this was not shown in the results section. Can't this be a sole reason why there is a disparity between male and female user activities shown?

The administrative actions show significant difference between the two genders while other actions show similar patterns; therefore, we believe that this can only lead to the conclusion that administrative tasks are gender oriented.

9. You will need an English speaker to read through this work to improve the flow especially with sentence starters.

Done

10. This is an interesting piece of work. Well done for the hard work.

Thank you very much for the valuable comments which highlighted important missing parts.

Reviewer #2: N/A, N/A, N/A, N/A, N/A, N/A, N/A, N/A, N/A, N/A, N/A, N/A, N/A, N/A, N/A, N/A, N/A, N/A, N/A, N/A, N/A, N/A, N/A, N/A, N/A, N/A, N/A, N/A, N/A, N/A, N/A, N/A, N/A, N/A, N/A, N/A, N/A, N/A, N/A, N/A, N/A

Reviewer #3: Method section is not clear, what software was used to analysis the data. I am confused about this section. Why is there no discussion to show similar work and how the findings relate to that work? the Conclusion needs a lot of work it is not very scientific and doesn't reflect much of the results.

We went through the manuscript to fix the writing issues, and we hope it looks better now. Thank you for your comments.

6. PLOS authors have the option to publish the peer review history of their article ([what does this mean?](https://journals.plos.org/plosone/s/editorial-and-peer-review-process#loc-peer-review-history)). If published, this will include your full peer review and any attached files.

If you choose “no”, your identity will remain anonymous but your review may still be made public.

**Do you want your identity to be public for this peer review?** For information about this choice, including consent withdrawal, please see our [Privacy Policy](https://www.plos.org/privacy-policy).

Reviewer #1: **Yes:** Olivia Nakisita

Reviewer #2: **Yes:** Daniel O'Keefe

Reviewer #3: No
