# Supplementary material for: Arabic Wikipedia users’ personalized behavior analysis considering gender gap
Source: PLoS One. 2024 Oct 21;19(10):e0312176. doi: 10.1371/journal.pone.0312176 (PMC11493248; doi:10.1371/journal.pone.0312176)
Supplement: Attachment — Submitted filename: Reviewers Comments.docx [file pone.0312176.s002.docx]

Reviewer #1:

1. The abstract should be presented (Introduction, Objective, Methods, Results, Conclusion) The method used was not well explained

**An abstract with the requested structure was included for the editor to choose which is better to include in the paper. Both abstracts contain the same information.**

2. The content with the heading of "Related Work" could be used for the discussion of results which you did not include in this manuscript

**Some parts have been used in this modified version.**

3. You indicated "Experimental Results" but you did not include experiments in your methodology. Review this and be specific. downloading information is not an experiment.

**The phrase was modified to “results” only**

4. Please include a "Discussion section" to this paper.

**There is a section named “Results and Discussions” starting line 175.**

Reviewer #2:

This version of the paper draft is a large improvement on the original. Well done. However, I feel there remains some work to be done before the paper can be published.

- The aims of the paper do not properly reflect the work performed, and are overcomplicated. Better to just specify that your aims were to (for example), "Describe the proportions of males and females among Arabic Wikipedia contributors".

**The study is not about proportions only, it can characterize behaviour, roles, and dedication on Wikipedia.**

- In the methods, you say "Wikipedia pages where usernames were matched to users found in history pages". The process for making this matching isn't properly explained. Is this all publicly available data? Do Wiki creators provide additional personal information about themselves (aside from gender)?

**It was stated in the last paragraph before the conclusion section, lines 263 and 264, that : “… however, since bibliographical data for Wikipedians are not available, it is impossible to confirm these assumptions …”**

**The matching process is now explained in Figure 1 and page edit history schema in Table 2. In line 157: “Contributors’ ids were used to match page contributors with their Wikipedia page edit history files.”**

- You haven't explained any of your statistical methodology in the methods section.

**All statistical methods used were basic methods, the most important part was combining this big data from Wikipedia and collect the statistics with their corresponding users and actions.**

- What is meant by "Page actions", "Revision actions" and "User actions" is not clear. Also, how does the same action (i.e. "page create") differ across each action?

**Actions were detailed in Table 3 in the supporting materials section at the end of the paper. If the reviewers feel that the table is not enough we will gladly explain them more.**

- There's a lot of information in the results section that should be moved to the discussion. For example, the 'gender analysis' would be better placed in the discussion (though needs citations).

**Some parts were modified and moved. This was an excellent suggestion, thank you.**

- Table 1 doesn't include any p-values.

**p-value was mentioned in the text. Nevertheless, p=0.05 was used.**

- As above, the discussion is incredibly short. This is your space to interpret your results and consider your results in relation to existing global literature.

**Done.**
