# Supplementary material for: Arabic Wikipedia users’ personalized behavior analysis considering gender gap
Source: PLoS One. 2024 Oct 21;19(10):e0312176. doi: 10.1371/journal.pone.0312176 (PMC11493248; doi:10.1371/journal.pone.0312176)
Supplement: Attachment — Submitted filename: Reviewer comments cycle #3 June 2024.docx [file pone.0312176.s003.docx]

6. Review Comments to the Author

Please use the space provided to explain your answers to the questions above. You may also include additional comments for the author, including concerns about dual publication, research ethics, or publication ethics. (Please upload your review as an attachment if it exceeds 20,000 characters)

**Reviewer #1:** The Authors have been able to address all the comments I raised. Their work is technically sound and should therefore be considered for publication.

**Thank you very much for the valuable comments. It made our manuscript looks better.**

**Reviewer #2:** Unfortunately, I've found the authors responses to my previous comments to be mostly insufficient - and in some cases, I'm more uncertain that I was previously. I return to my previous comments, and hope for more comprehensive response:

1) While you may have aimed to characterise actions performed on Wikipedia according to gender, this has only been done descriptively, using basic proportions. Even so, my previous issue was that this relatively basic aim was stated across eight separate aims, some of which are largely repeating themselves. The current eight aims could simply be summarised using a single aim, such as "We aimed to describe the interactions with Wikipedia pages across time, according to gender". Beyond something like this, I think you may be overselling the abilities of your analysis methods.

**The issue was previously addressed in Line 90 as follows: “This study aims to explore the behaviour of Arab users of Wikipedia, with an emphasis on gender in relation to contributions, resources, and time dedicated to their involvement”**

**Afterwards, the aim was split over various research questions covering contribution size (Q2), contribution types (Q3), contribution date and time (Q4), contribution topics of interest (Q5, 6, 7), and contribution changes over the past 5 years (Q8). In addition, it was interesting to study the inverse relationship between contributions and gender (Q1).**

**All the questions were addressed within the text, the tables, and the figures.**

2) Based on the authors response, unfortunately, I'm now more confused by the matching process. The authors point to Figure 1, as an example of the metadata extracted - where I can't see any mention of gender specification. The authors also point to a statement in the discussion, being "...since bibliographic data for Wikipedians..." - the context of this sentence suggests you mean "biographic" rather than "bibliographic" - is this what you meant? If so, any biographic data would include gender...

Either way, the methods still don't adequate describe how the authors have extracted user data. Currently, it reads that a user name has simply been matched across pages using metadata - but how this has been used to determine gender is not explained. The wording on page 9, and the reference to citation 28 are not helpful in this regard. Can you please ensure you are simply, but explicitly, explaining the process you used here.

**True, it is biographical data as fixed in line 264 “however, since biographical data for Wikipedians…”, and to make sure it is clear, Wikipedia does not reveal personal information about their users due to privacy issues.**

**The matching process is described in the text as follows: “Therefore, a list of Arabic men and women users have been collected from the ( تصنيف:رجال_ويكيبيديون ، تصنيف:نساء_ويكيبيديات translated in [28]) Wikipedia pages where usernames were matched to the users found in history pages, and the matched ones were reported in this work”**

**To elaborate, if you go to Wikipedia, and open the categories listed in reference 28 you will find a list of Wikipedia male and female users in the two categories mentioned in the reference. So basically, the only way we could tell whether a user is male or female is by the category pages of Wikipedia listing them as males or females. Following a sample from the male category page showing that user: Ammar2010 (for example) is a male user.**

3) The use of "basic" statistical methods does not preclude you from providing detail of your methods in the paper. You repeatedly refer to a "significant" difference in your results section, but without prior description of your analytical methods. For example, you refer to a "significant" difference in Figure 2 - so, how did you perform this trend analysis?

**Fixed. With all significance mention, the method (e.g. t-test) and alpha value were mentioned.**

4) I fee like short definitions of what is meant by your "page" and "revision" categories is warranted in the methods section. It is currently unclear, and a reader shouldn't have to go hunting in the supplementary material for this information. Further, now seeing the definitions, I wonder what is the relevance of checking gender against users creating or renaming accounts??

**We understand that the schema of Wikipedia should be in the text; however the result will be the same since tables are separated from the text, therefore we felt that the location of the schema is not important; nevertheless, if the journal decides it is important to add the table reference inside the text, we will do it.**

**With reference to your other part of the comment, “I wonder what is the relevance of checking gender against users creating or renaming accounts??” This resembles an indicator on what privileges the users may have, since editing and naming/renaming users is a high level authority. Matching this with gender can show whether there is a difference in authority roles between the two genders. This was concluded in the abstract line 32**

5) The reason I asked for p-values in Table 1 is because the text currently suggests that all means across categories were significant. Is this accurate? I repeat that it would be a good idea to state all relevant p-values.

**Alpha value was added as answered in comment 3.**
