# Supplementary material for: Arabic Wikipedia users’ personalized behavior analysis considering gender gap
Source: PLoS One. 2024 Oct 21;19(10):e0312176. doi: 10.1371/journal.pone.0312176 (PMC11493248; doi:10.1371/journal.pone.0312176)
Supplement: Attachment — Submitted filename: Reviewer comments cycle #4 Sept 2024.docx [file pone.0312176.s004.docx]

**Dear Reviewers,**

**Thank you very much for your comments. We tried to accommodate your comments as possible. We hope that this version of the paper will get your approval for publication. Please find our answers to your comments in RED.**

**Best Regards,**

**Bashar, on behalf of the authors**

**Reviewer #1:**

1. The abstract is now well structured however the conclusion is written more as the results and the results more as the conclusion. The methodology should clearly state how you got the data you used for this study.

DONE

2. The subheading "Related Work" should be added to the introduction

It is not possible to do that because this paper includes professors from different schools, and since it maybe acceptable to do this in one domain it is not possible to have a paper without related work section.

3. There are no results tables to support the association statistics provided

We have not provided any association data or conclusions based on association in the paper, otherwise all statistics are provided within the supported materials.

4. The results and discussion section should be presented independently and not combined

In our paper, it is important to comment on each result table/figure directly after it was listed. This is also a common practice in the domain of IT.

Thank you very much for putting the effort to help us enhance the quality of our research. We really appreciate your reviews every time.

**Reviewer #4:**

 The manuscript is properly revised, and sufficiently improved. I think the manuscript is ready for publication.

Thank you very much for helping us enhance the quality of our research.
